# Presence of Obsessionality and Hysteria in Patients with Primary Open-Angle Glaucoma

**DOI:** 10.3390/medicina62081510

**Published:** 2026-08-06

**Authors:** Marija Olujić, Slaven Balog, Stipe Vidović, Ivana Kotromanović Šimić, Darko Kotromanović, Dubravka Biuk, Katarina Dodig-Ćurković

**Affiliations:** 1 Ophthalmology Polyclinic Dr. Balog, Ivana Gundulića 36 b, 31000 Osijek, Croatia; slavenbalog@gmail.com; 2 Faculty of Medicine Osijek, Josip Juraj Strossmayer University of Osijek, Josipa Huttlera 4, 31000 Osijek, Croatia; ivana.simic.osijek@gmail.com (I.K.Š.); kotromanovic93@gmail.com (D.K.); katarina5dodig@gmail.com (K.D.-Ć.); 3 Clinic for Eye Diseases, Clinical Hospital Center Osijek, Europska Avenija 14, 31000 Osijek, Croatia; stipevidovic1@gmail.com; 4 Oncology Clinic, Clinical Hospital Center Osijek, Josipa Huttlera 4, 31000 Osijek, Croatia; 5 Institute for Child and Adolescent Psychiatry, Clinical Hospital Center Osijek, Europska Avenija 14, 31000 Osijek, Croatia; 6 Faculty of Dental Medicine and Health Osijek, Josip Juraj Strossmayer University of Osijek, Crkvena 21, 31000 Osijek, Croatia

**Keywords:** glaucoma, open-angle, hysteria, intraocular pressure, mental disorders, mental health, obsessive behavior, quality of life, stress, psychological, surveys and questionnaires

## Abstract

*Background and Objectives*: Glaucoma is a group of conditions of the eye that can damage the optic nerve, leading to optic neuropathy. Nevertheless, due to its chronic characteristics, it could also be the background for psychoneurotic disorders based on the psychological and emotional stress that it causes. The presented study aimed to examine the characteristics and the occurrence of obsessionality and hysteria, as well as Open-Angle Glaucoma (OAG) in glaucoma patients. *Materials and Methods*: A cross-sectional study was conducted in the Clinic for Eye Diseases at the Clinical Hospital Center Osijek on 200 patients with different stages of OAG progression from December 2023 to December 2024. A Crown–Crisp Experiential Index (CCEI) was used to observe the influence of different degrees of OAG damage on the severity of obsessionality and hysteria. *Results*: The obsessionality score was higher in women than in men (Mann–Whitney U test, *p* < 0.001). Multivariate logistic regression (stepwise method) indicated a significant model for predicting hysteria, consisting of one predictor: female sex (odds ratio = 2.9). According to CCEI, using bivariate and multivariate logistic regression to predict obsessionality, no significant predictors or models were observed. *Conclusions*: The psychological toll of coping with glaucoma could exacerbate glaucoma. Further investigations are necessary to help us understand OAG personality traits and establish a stable methodology for extending appropriate care to glaucoma patients.

## 1. Introduction

Glaucoma is considered a chronic, progressive disease characterized by the progressive deterioration of the optic nerve fibers and the associated retinal nerve fibers. Owing to the above, characteristic visual field (VF) defects occur [1]. This also leads to deterioration in the quality of life of glaucoma patients. High or elevated levels of intraocular pressure (IOP) are still considered the main risk factor for glaucoma. Moreover, it remains the only risk factor that can be influenced by anti-glaucoma therapy (AGT) [2]. Glaucoma is the cause of vision loss in tens of millions of people worldwide. It affects an average of 2% of the population over 40 years of age, and the impaired vision causes deteriorated quality of life in affected patients [3]. The global prevalence for the population between 40 and 80 years of age is estimated at 3.5% [4]. As the world population ages, the prevalence of glaucoma is increasing. It is also predicted that an increasing number of the elderly population will suffer from glaucoma in the future, and according to current estimates, 111.8 million people will suffer from glaucoma of all types by 2040 [4]. According to the above, this would result in bilateral blindness estimated in 10% of all glaucoma patients worldwide or significant bilateral VF loss [5,6]. Elevated IOP is currently the only risk factor that could be effectively modified [2]. Nevertheless, patients first face the fact that there may be a gradual deterioration of vision and VF defects that may progress over the years of the disease, which could ultimately lead to blindness [6].

The relationship observed between glaucoma and stress is becoming a topic of interest and importance and could be the key for determining the disease risk in vulnerable individuals. Screening for glaucoma in individuals exposed to high stress and targeted interventions could be beneficial in preserving vision and improving outcomes for those diagnosed with glaucoma [2]. It is more pronounced in those who suffer from chronic Primary Open-Angle Glaucoma (POAG) and usually includes signs of neuroticism, along with present hypochondriac tendencies, as well as tendencies towards perfectionism, but also introversion, conscientiousness, and further meticulousness [7,8]. A five-factor model could be used to perform accurate personality evaluation [9], which could help the glaucoma specialist gain quick insight into the psychological state of their patients.

It is of the utmost importance to establish a healthy and professional relationship between the patient and glaucoma specialist in order to notice and react in a timely manner to any changes in personality traits of patients. Moreover, this is the only way to provide timely psychological support for glaucoma patients since they have been experiencing not only pressure from the fact that they are suffering from a life-time chronic disease, but also from the fact that it is a progressive disease, meaning their clinical state could worsen. However, they may have been using AGT medications properly as prescribed to them and, nevertheless, still experience a constant fear of blindness. If glaucoma is not treated on time, it can lead to vision loss and eventually lead to a terminal state—blindness [6].

Even though it is estimated that in Western countries half of all glaucoma patients are undiagnosed or often diagnosed too late [6], the number of glaucoma patients still tends to increase, and Croatia is not an exception. The latest available data from primary healthcare from 2012 show that there were almost 70,000 cases of glaucoma in Croatia at that time [10]. In Osijek-Baranja County, according to the latest available data from a study from 2017, a total of 5.6% of the population suffered from Open-Angle Glaucoma (OAG) [11].

The conducted study aimed to examine and evaluate the characteristics of the occurrence of obsessionality, hysteria, and OAG in patients.

Previously, in Croatia, there was a study considering anxiety and depression in glaucoma patients [12]. Nevertheless, there is no research conducted on this topic; therefore, this is the first study in Croatia that has observed psychoneurotic disorders of OAG patients, regarding hysteria and obsessionality. The lack of literature on this topic points to the value of this study because it bridges the gap in the described area.

## 2. Materials and Methods

### 2.1. Study Approval

The Ethics Committee of the Clinical Hospital Center Osijek approved this study (approval date: 11 November 2022, Number: R1-14554/2022). Moreover, the Ethics Committee of the Faculty of Medicine Osijek also approved this study (approval date: 23 February 2023, Class: 602-04/23-08/03, Reg. Number: 2158-61-46-23-17). Informed consent for voluntary participation in the study has been obtained from all eligible patients who met the appropriate inclusion criteria. The complete methodology of this cross-sectional study, as well as the investigated factors, was described in a previous study [12].

### 2.2. Participants

Two hundred patients diagnosed with POAG of three different stages of severity were included in this study. The study population consisted of 68 (34%) men and 132 (66%) women, resulting in a predominance of female participants. The study was conducted at the Glaucoma Infirmary of the Clinic for Eye Diseases of the Clinical Hospital Center Osijek, from December 2023 to December 2024. Participants were included if they had POAG, were treated with topical AGT, and were aged 18 to 70 years. On the other hand, exclusion criteria included age over 70 years, prior glaucoma surgery or iridotomy, or other diagnoses except POAG. The severity of the POAG was determined based on the extent of VF damage, as well as pertinent structural metrics of the optic nerve head. These assessments are a part of the routine clinical evaluation and/or eligibility assessment of the participants [6]. Optical coherence tomography (OCT) was performed using the REVO 60 spectral-domain OCT (OPTOPOL Technology Sp. z o.o., Zawiercie, Poland; SOCT software version 11.5.0), and standard automated perimetry was performed using the Octopus 900 perimeter (Haag-Streit AG, Köniz, Switzerland; EyeSuite software version i9.3.0).

Of the initial 250 patients assessed for eligibility, 33 were excluded because they did not meet the inclusion and exclusion criteria. Of the remaining 227 eligible patients, 27 declined to participate. Consequently, 200 patients were enrolled in the study, corresponding to a participation rate of 88.1%. All enrolled participants completed the questionnaire without missing data, resulting in a 100% questionnaire completion rate.

Participants in this study underwent a complete ophthalmological examination. A comprehensive medical history was taken; a slit-lamp biomicroscope was used for anterior and posterior segment examination; and IOP was measured with a ‘gold standard’ Goldmann applanation tonometry. Participants also completed the Crown–Crisp Experiential Index (CCEI) questionnaire, along with recording general information.

### 2.3. The Crown–Crisp Experiential Index Questionnaire

The CCEI questionnaire was essential for this study. The results of this questionnaire could identify and measure the most frequent symptoms of the patients, as well as their personality traits within the usual categories, pointing to psychoneurotic diseases as well as personality disorders. Psychoneurotic disorders are usually listed within six subscales as follows: obsessionality, hysteria, depression, free-floating anxiety, phobic anxiety, and somatic manifestations of anxiety. The total score provides a measure of general emotional instability or neuroticism with a profile of six subscale scores [13,14]. The obtained version of the CCEI questionnaire was translated into the Croatian language and published by “Naklada Slap”. “Naklada Slap” has previously given its written permission to use 200 copies of the CCEI questionnaire to be able to conduct this research. The aforementioned CCEI has previously undergone linguistic and cultural validation processes.

For logistic regression analyses, the obsessionality and hysteria subscales were dichotomized according to the normative values of the Croatian version of CCEI (Table 1). Age- and sex-specific normative values published in the CCEI manual were used as reference values. Scores exceeding the corresponding normative value were classified as “Yes” (expressed obsessionality/hysteria), whereas scores within the normative range were classified as “No”.

Along with the CCEI questionnaire, the following information was gathered: participants’ sex and age, severity of POAG damage (stage), how long the POAG was treated, daily applied topical AGT (number), if the patient has achieved the target IOP with AGT (yes/no), other ophthalmological conditions before the diagnosis of POAG (yes/no), and feeling of stress (yes/no).

This study has provided observation of the associations between the variables of interest without interfering with the medical treatment.

### 2.4. Statistical Methods

G*Power (ver. 3.1.2.) was used to determine the minimum required sample size necessary for three independent groups of participants. The significance level was set at 0.05, with power set at 0.80. Moreover, statistically, the minimum required sample size was determined to be 159 subjects. Furthermore, the minimum causal size was determined as 159 subjects, or 58 participants per group.

Categorical data were presented in two ways, as absolute and relative frequencies. Differences in categorical variables were tested with the χ^2^ test and, when necessary, Fisher’s exact test was used. The Shapiro–Wilk test was used to test the normality of the distribution of continuous variables. Continuous data were presented as the median and interquartile range (IQR). The Mann–Whitney U test was used for testing the differences in continuous variables between two independent groups (the Hodges–Lehmann difference in median with the corresponding 95% confidence interval (CI)). The Kruskal–Wallis test (with the Conover post hoc test) was used for testing the differences in continuous variables between three independent groups. As the distributions of the CCEI subscale scores deviated from normality according to the Shapiro–Wilk test, non-parametric methods were applied for group comparisons. Spearman’s rank correlation coefficient (ρ) was used to assess the strength and direction of monotonic associations between continuous or ordinal variables. Binary logistic regression analyses were performed to identify factors associated with expressed obsessionality and hysteria (Yes/No). Variables that were clinically relevant or examined in the bivariate analyses (sex, age, glaucoma stage, subjective stress, target IOP achievement, duration of treatment, and medication use) were entered into the regression models. Odds ratios (ORs) with corresponding 95% confidence intervals (CIs) were reported [15,16]. All values were two-sided. The significance level was set at alpha = 0.05. For statistical analysis, the statistical package MedCalc^®^ Statistical Software version 23.1.1 was used [17]. The report on the conducted research was prepared according to previously established guidelines for reporting the results of research in biomedicine and healthcare [18].

## 3. Results

### 3.1. Basic Characteristics of Participants

The presented study was conducted on 200 participants, treated for POAG over the 2023 to 2024 period [68 (34%) men and 132 (66%) women]. Most participants were 56 to 65 years of age. Participants were equally distributed across all three degrees of POAG. Prior ophthalmological diseases have been recorded in 36 (18%) participants. The most common comorbidity was arterial hypertension in 135 (67.5%) participants, and 57 (28.5%) participants had diabetes, of whom only four (7%) had type 1. A total of 93 (46.5%) participants had experienced a feeling of stress (Table 2).

The median age of the participants in this study was 65 years (range: 37 to 70 years). The median duration of POAG treatment was 96 months (8 years) [range: 1 month to 564 months (47 years)]. AGT was generally prescribed from 1 time/day to 4 times/day (median: 2 times/day).

### 3.2. Correlation of Obsessionality Subscale with Characteristics of Participants

The obsessionality score was significantly higher in women than in men (Mann–Whitney U test, *p* < 0.001), in participants who reported feeling stressed, compared to those who did not feel this way (Mann–Whitney U test, *p* = 0.005), as well as in participants who achieved the target values of the IOP (Mann–Whitney U test, *p* = 0.01). At the same time, there were no significant differences in the score of the obsessionality subscale according to other characteristics of the participants (Table 3).

There were no significant differences in the distribution of participants concerning the severity of the obsessionality subscale (Table 4).

### 3.3. Correlation of the Hysteria Subscale with the Participants’ Characteristics

In the hysteria subscale, there was a significantly higher score in women compared to men (Mann–Whitney U test, *p* < 0.001), and in participants who stated that they felt stressed, compared to those who did not (Mann–Whitney U test, *p* = 0.04). At the same time, there were no significant differences in the hysteria subscale score according to the other characteristics of the participants (Table 5).

The pronounced hysteria subscale was significantly higher in women than in men (χ^2^ test, *p* = 0.005). On the other hand, no significant differences in the distribution of participants according to the severity of hysteria were noticed in the other characteristics (Table 6).

### 3.4. Association of Psychoneurotic Disorders (CCEI Questionnaire) with the Age of the Participants, the Duration of Treatment and the Degree of POAG

Spearman’s correlation coefficient was used to assess the correlation between participants’ age, treatment duration, and the degree of glaucoma with the subscales of the CCEI questionnaire. It is observed that the significant correlations are somewhat weaker (Rho < 0.5).

Within the group of all participants, the lower the stage of glaucoma, the more enhanced the obsessionality (Rho = −0.147). In the group of participants who did not achieve the target IOP, participants with a more pronounced stage of glaucoma have a less enhanced hysteria subscale (Rho = −0.323) (Table 7).

In predicting the expression of obsessionality according to the CCEI, no significant predictors or models were observed in bivariate and multivariate logistic regression (Table 8).

In the prediction of hysteria according to CCEI, in bivariate logistic regression, it was observed that only the female sex is a significant predictor of hysteria.

Multivariate logistic regression (stepwise method) showed that there is a significant model in the prediction of hysteria, and it is made up of one predictor: female sex (OR = 2.9). The overall multivariate logistic regression model was statistically significant (χ^2^ = 8.5, *p* = 0.004). Female sex remained the only independent predictor of expressed hysteria (OR = 2.9, 95% CI 1.35–6.22) (Table 9).

## 4. Discussion

This study examined the occurrence of obsessionality and hysteria in patients with OAG of different degrees of damage. It has also examined the characteristics of glaucoma patients with signs of obsessionality and hysteria, as well as those without pronounced features of the aforementioned psychoneurotic disorders, by sex, age, and type of AGT they use. The results obtained in the study suggest that, in patients with OAG, obsessionality and hysteria occur, depending on the stage of progression of glaucomatous damage. Although female sex emerged as the only significant predictor of hysteria in this study, this finding should be interpreted cautiously because women were substantially overrepresented in the study sample. Nevertheless, the model explains only a small proportion of the observed variability. This finding suggests that additional biological, psychological, and social factors not included in the present study are likely to contribute to hysteria scores in patients with POAG. Accordingly, these results should be interpreted with appropriate caution. Moreover, this study is the first to demonstrate an association between observed psychoneurotic disorders (obsessionality and hysteria) in OAG patients in Croatia.

Quality of vision is a necessary and integral part of everyday life and functioning in the environment. When quality of vision is preserved, it is easier to function and interact with one’s surroundings. A person with good vision can move in space, navigate it, and be independent [19]. Damage to an individual’s vision, depending on its extent, can have long-term consequences on the quality of life, but it can also have psychological repercussions. This condition can lead to an inability to perform the job the patient previously did, which can cause financial instability and insecurity and the need to change occupations. Also, because of the narrowing of the field of vision, accidents can occur (the patient may accidentally get hit, fall, and/or get injured), which can also lead to temporary incapacity for work and weaker financial power. For those patients who already have weakened financial capabilities, the prescription of topical AGT drops, which are paid for additionally, could cause additional financial issues. Each of the above factors can lead to the development of stress and behavioral changes in glaucoma patients [8] and precede the development of psychoneurotic disorders, usually anxiety and depression [12]. Although obsessionality and hysteria have not been previously examined in relation to glaucoma patients, this study also demonstrated a potential association.

Even though patients mostly adhere to the recommended guidelines for the use of prescribed AGT, because of the progressive nature of the disease, glaucoma can continue to progress and lead to the most severe outcome: blindness. Some patients, in such circumstances, voluntarily give up the recommended AGT, which increases the risk of additional deterioration of the clinical condition of their optic nerve. Because of the reduction in adherence to the prescribed therapy, i.e., reduction in the patient’s cooperation with the applied therapy, the patients enter the “vicious cycle” within which they further deepen and worsen glaucomatous eye diseases and thus, as a result, impair their psychological state. It can also disrupt their financial situation, leading to stress. Therefore, stressful situations could have a significant impact and enhance the risk for development and progression of OAG—which could lead to elevation of the IOP and progression of glaucomatous disease. Along with the most common personality traits in glaucoma patients (anxiety and depression), others that are stress-related are also encountered (e.g., sleep disorders, psychotic disorder, and dementia, as well as post-traumatic stress disorder) [2].

In a study conducted by Lim et al., an increased prevalence of hypochondria, hysteria, and health problems in patients with OAG was observed. Those patients also had a significantly higher clinical abnormal score for hysteria and health problems, which was also associated with the number of medications they consumed [20]. In the context of psychoneurotic disorders that Lim et al. found in their study, we also observed a similar finding, i.e., that people suffering from chronic diseases such as glaucoma, due to their health problems, may have a more pronounced neurotic personality trait, which leads to the development of symptoms of hysteria, but also obsessionality. This could also be the reason why they are more committed to prescribed AGT.

Mabuchi et al. also studied the characteristic personality traits in patients with POAG and have found that the association was somewhat more pronounced in men, in contrast to the results presented here. In fact, they additionally found signs of neuroticism, symptoms of hypochondria, and patients’ tendency to be introverted. On the other hand, they are very conscious as patients, meticulous, and prone to perfectionist behavior (which is extremely useful in the application of AGT in an adequate manner) [9].

When treating glaucoma patients, it is difficult to motivate patients to persist in prescribed adequate AGT [21]. AGT should be tailored individually for each patient, primarily based on the current state of their disease and their characteristics—both personality and cooperation. The decision of the competent ophthalmologist should be clearly and understandably communicated to each patient to benefit the patient, i.e., to maintain and preserve the existing glaucoma damage for as long as possible. Of course, it would be necessary to periodically assess the patient’s psychological state to timely recognize the development of a psychological disorder that would lead to premature abandonment and discontinuation of the recommended therapy.

Given the diversity of therapeutic approaches, collaboration and communication between ophthalmologists and patients are necessary to find the best solution that will maintain the patient’s motivation and desire to continue treatment and slow the deterioration of the optic nerve. A holistic and multidisciplinary approach between patients and ophthalmologists in cooperation with psychiatrists and psychologists [8], non-pharmacologic methods of stress reduction and relaxation techniques [2], along with an adequate multidisciplinary approach (interaction between glaucoma specialists and psychiatrists), could potentially be a solution to allow the patient to preserve the existing eye condition for as long as possible.

Nevertheless, this study has some limitations that could affect the reliability of its results. The cross-sectional design of this study prevents the establishment of causality. Accordingly, only associations between the observed variables could be identified. Despite including 200 participants, the relatively small, convenience-based sample may limit the generalizability and accuracy of the results. Furthermore, women represented approximately two-thirds of the study population, resulting in an imbalanced sex distribution. This may have influenced the observed associations, particularly those related to sex differences in obsessionality and hysteria, and therefore these findings should be interpreted with caution. Future studies should aim to recruit a more balanced sample with respect to sex. A recommendation for future studies would also be to include appropriate control groups to better distinguish glaucoma-specific psychological characteristics from those associated with chronic disease in general. Moreover, the duration of the study itself and data collection time were relatively short, which may also limit the accuracy of the study results. The study’s focus on a single tertiary institution may also limit the generalization of the findings. Also, a 100% response rate may reflect characteristics of the single-center study setting and limit the applicability of our findings to broader glaucoma populations. Lastly, self-report questionnaires could limit the study because participants might respond based on their current emotions, not their actual clinical state. This could disturb and complicate the interpretation of the results.

Further research would be advisable, preferably designed as a longitudinal or even interventional study. This approach would clarify the causality of the observed variables and the relationship between psychoneurotic disorders and patients with POAG. Additional research is required to elucidate the relationship between personality types and characteristics, chronically present stress factors, and the risk of OAG onset and progression. It would also be necessary to continue with further investigations to move from an understanding of POAG personality traits toward establishing a methodology for appropriate care of glaucoma patients, with a tendency to improve their quality of daily life.

## 5. Conclusions

The results indicate the need to design targeted interventions for glaucoma patients with behavioral changes and suspected development of psychoneurotic disorders. Looking at the assessment of obsessionality based on the CCEI, it is significantly higher in women compared to men, in respondents who report feeling stressed, compared to the ones reporting the opposite, as well as in respondents who have achieved target values of the IOP; nevertheless, it is important to emphasize that the sample population was skewed towards the female population. On the other hand, in the prediction of hysteria according to CCEI, in a bivariate logistic regression, it is observed that only female sex is a significant predictor of hysteria. Future studies including a more balanced representation of male and female participants are warranted to validate these findings.

Through timely recognition of behavioral changes in glaucoma patients (e.g., using short questionnaires during ophthalmological check-ups) and targeted interventions, glaucoma specialists could suspect that the patient is developing a psychoneurotic disorder that could lead to changes in the patient’s adherence to AGT. Finally, since local AGT is based on the trust of the physician and patient that the patient is indeed adequately using the prescribed therapy, if patients independently decide to stop using the prescribed AGT, this could ultimately lead to a deterioration of the clinical condition. Therefore, an adjacent holistic, multidisciplinary approach (between glaucoma specialist, psychiatrist and psychologist), as well as a non-pharmacological approach, as previously described, could be a potential way to respond in a timely manner and improve the quality of care for glaucoma patients and preserve their current quality of life for as long as possible.

## Figures and Tables

**Table 1:** CCEI standard interval values for determining the presence of psychoneurotic disorders of hysteria and obsession in participants, depending on sex and age group.

	Hysteria		Obsessionality	
Age Groups	Women	Men	Women	Men
35–44	6.7 ± 3.1	6.9 ± 2.9	4.2 ± 2.9	3.2 ± 2.5
45–54	7.5 ± 3.0	7.2 ± 2.9	3.6 ± 2.8	2.6 ± 2.6
55–64	7.3 ± 2.7	7.6 ± 2.8	3.0 ± 2.8	3.0 ± 2.7
65–74	7.5 ± 2.7	7.1 ± 3.1	2.4 ± 2.5	2.6 ± 2.9

**Table 2:** General and clinical characteristics of the participants.

	Number (%) of Participants
Sex	
Men	68 (34)
Women	132 (66)
Age groups	
<55 years	22 (11.0)
56–65 years	92 (46.0)
>66 years	86 (43.0)
OAG stage	
I	68 (34.0)
II	65 (32.5)
III	67 (33.5)
Pre-existing ophthalmological diseases	36 (18.0)
Comorbidities	
Diabetes	57 (28.5)
Type of diabetes (*n* = 57)	
Type 1	4 (7.0)
Type 2	53 (93.0)
Arterial hypertension	135 (67.5)
Subjective feeling of stress	93 (46.5)
Target intraocular pressure (IOP) achieved	150 (75)

**Table 3:** Obsessionality subscale score in relation to participants’ characteristics.

	Median (IQR) Obsessionality	Difference	95% CI	*p* *
Sex				
Men	7 (5–9)	2	1 to 2	**<0.001**
Women	9 (7–10)			
Age groups				
<55 years	8 (7–8)			0.19 ^†^
56–65 years	8 (6–10)	-	-	
>66 years	9 (7–11)			
OAG stage				
I	8 (7–11)			0.12 ^†^
II	8 (7–10)	-	-	
III	8 (5–10)			
Diabetes				
No	8 (6–10)	−1	−2 to 0	0.15
Yes	7 (6–10)			
Type of diabetes (n = 57)				
Type 1	9 (5–9)	0	-	0.98
Type 2	7 (6–10)			
Arterial hypertension				
No	8 (6–10)	0	−1 to 1	0.73
Yes	8 (6–10)			
Feeling stressed				
No	8 (6–9)	1	0 to 2	**0.005**
Yes	9 (7–11)			
IOP target values achieved				
No	7 (5–10)	1	0 to 2	**0.01**
Yes	8 (7–10)			

* Mann–Whitney U test; ^†^ Kruskal–Wallis test. Statistically significant values were written in bold.

**Table 4:** Severity of obsessionality in relation to characteristics of the participants.

	Number (%) of Participants According to the Severity of Obsessionality			*p* *
	No (*n* = 158)	Yes (*n* = 42)	Total (*n* = 200)	
Sex				
Men	58 (36.7)	10 (23.8)	68 (34)	0.12
Women	100 (63.3)	32 (76.2)	132 (66)	
Age groups				
<55 years	21 (13.3)	1 (2.4)	22 (11)	0.07
56–65 years	74 (46.8)	18 (42.9)	92 (46)	
>66 years	63 (39.9)	23 (54.8)	86 (43)	
OAG stage				
I	50 (31.6)	18 (42.9)	68 (34)	0.29
II	55 (34.8)	10 (23.8)	65 (32.5)	
III	53 (33.5)	14 (33.3)	67 (33.5)	
Achieved IOP target values	116 (73.4)	34 (81)	150 (75)	0.32
Feeling stressed	68 (43)	25 (59.5)	93 (46.5)	0.06
Diabetes	47 (29.7)	10 (23.8)	57 (28.5)	0.45
Arterial hypertension	106 (67.1)	29 (69)	135 (67.5)	0.81
Daily medication use	135 (85.4)	37 (88.1)	172 (86)	0.66
Antihypertensives	108 (80)	30 (81.1)	138 (80.2)	0.88
Antihyperglycemic agents	44 (32.6)	10 (27)	54 (31.4)	0.52

* χ^2^ test.

**Table 5:** Hysteria subscale score in relation to participants’ characteristics.

	Median (IQR) Hysteria Subscale	Difference	95% CI	*p* *
Sex				
Men	3 (1–4)	1	1 to 2	**<0.001**
Women	4 (3–6)			
Age groups				
<55 years	4 (3–5)			0.77
56–65 years	4 (2–5)	-	-	
>66 years	4 (2–6)			
OAG stage				
I	4 (2–7)			0.06
II	4 (3–5)	-	-	
III	3 (2–8)			
Diabetes				
No	4 (3–6)	−1	−1 to 0	0.14
Yes	4 (2–5)			
Type of diabetes				
Type 1	4 (3–7)	−1	-	0.40
Type 2	4 (2–5)			
Arterial hypertension				
No	4 (2–5)	0	0 to 1	0.28
Yes	4 (3–6)			
Feeling stressed				
No	3 (2–5)	1	0 to 1	**0.04**
Yes	4 (3–5)			
IOP target values achieved				
No	3 (2–5)	1	0 to 1	0.09
Yes	4 (3–6)			

* Mann–Whitney U test. Statistically significant values were written in bold.

**Table 6:** Expressiveness of the hysteria subscale in relation to the characteristics of the participants.

	Number (%) of Participants According to Severity of Hysteria			*p* *
	No (*n* = 146)	Yes (*n* = 54)	Total (*n* = 200)	
Sex				
Men	58 (39.7)	10 (18.5)	68 (34)	**0.005**
Women	88 (60.3)	44 (81.5)	132 (66)	
Age groups				
<55 years	18 (12.3)	4 (7.4)	22 (11)	0.09
56–65 years	72 (49.3)	20 (37)	92 (46)	
>66 years	56 (38.4)	30 (55.6)	86 (43)	
OAG stage				
I	48 (32.9)	20 (37)	68 (34)	0.21
II	44 (30.1)	21 (38.9)	65 (32.5)	
III	54 (37)	13 (24.1)	67 (33.5)	
Achieved IOP target values	107 (73.3)	43 (79.6)	150 (75)	0.36
Feeling stressed	66 (45.2)	27 (50)	93 (46.5)	0.56
Diabetes	43 (29.5)	14 (25.9)	57 (28.5)	0.62
Arterial hypertension	97 (66.4)	38 (70.4)	135 (67.5)	0.60
Daily medication use	125 (85.6)	47 (87)	172 (86)	0.80
Antihypertensives	99 (79.2)	39 (83)	138 (80.2)	0.58
Antihyperglycemic agents	41 (32.8)	13 (27.7)	54 (31.4)	0.52

* χ^2^ test.

**Table 7:** Correlation of age of participants, duration of treatment and degree of glaucoma with subscales of the CCEI questionnaire in all participants, and in groups according to achieved target IOP values.

	Spearman’s Rho Correlation Coefficient (*p* Value)		
	Age of the Participant	Duration of Treatment	OAG Stage
All participants			
Obsessionality	0.050 (0.48)	0.050 (0.48)	**−0.147 (0.04)**
Hysteria	−0.021 (0.77)	0.051 (0.48)	−0.134 (0.06)
Target IOP was not achieved			
Obsessionality	−0.057 (0.69)	0.013 (0.93)	−0.104 (0.47)
Hysteria	−0.125 (0.39)	−0.086 (0.55)	**−0.323 (0.02)**
Achieved IOP target values			
Obsessionality	0.096 (0.24)	0.068 (0.41)	−0.094 (0.26)
Hysteria	0.016 (0.84)	0.100 (0.22)	−0.028 (0.74)

Statistically significant values were written in bold.

**Table 8:** Prediction of expressed obsessionality according to the CCEI questionnaire (bivariate and multivariate logistic regression).

Obsessionality	β	Wald	*p*	OR	95% CI
Bivariate regression					
Sex (Women)	−0.62	2.41	0.12	0.54	0.25 to 1.18
Age	0.03	1.26	0.26	1.03	0.97 to 1.09
Age groups (<55 years)					
56–65	1.63	2.38	0.12	5.11	0.64 to 40.5
>66 years	2.04	3.75	0.05	7.67	0.98 to 60.3
OAG stage (I)					
II	−0.68	2.41	0.12	0.51	0.21 to 1.20
III	−0.31	0.58	0.45	0.73	0.33 to 1.63
Feeling stressed	0.67	3.56	0.06	4.95	0.97 to 3.88
IOP target values	−0.43	0.99	0.32	0.65	0.28 to 1.52
Duration of treatment	−0.01	0.09	0.77	0.99	0.95 to 1.04
Medication use	0.23	0.19	0.66	1.26	0.45 to 3.54
Multivariate regression					
No significant pattern					

β—regression coefficient.

**Table 9:** Prediction of hysteria according to the CCEI questionnaire (bivariate and multivariate logistic regression).

Hysteria	β	Wald	*p*	OR	95% CI
Bivariate regression					
Sex (women)	1.07	7.49	**0.006**	2.9	1.35 to 6.22
Age	0.04	2.23	0.14	1.04	0.99 to 1.10
Age groups (up to 55)					
56–65	0.22	0.13	0.71	1.25	0.37 to 4.11
66 and over	0.89	2.17	0.14	2.41	0.75 to 7.77
OAG stage (I)					
Stage II	0.14	0.13	0.72	1.15	0.55 to 2.39
Stage III	−0.55	1.81	0.18	0.58	0.26 to 1.28
Feeling stressed	0.19	0.36	0.55	1.21	0.65 to 2.26
IOP target values	−0.35	0.84	0.36	0.70	0.33 to 1.50
Duration of treatment	−0.001	0.003	0.98	0.99	0.96 to 1.04
Medication use	0.12	0.07	0.80	1.13	0.45 to 2.83
Multivariate regression					
Sex (women)	1.06	7.48	**0.006**	2.9	1.35 to 6.22
*Constant*	−2.82	15.8	**<0.001**		

β—regression coefficient. Statistically significant values were written in bold.

## Data Availability

All data are available and can be delivered to anyone upon request.

## Abbreviations

The following abbreviations are used in this manuscript:

AGT	anti-glaucoma therapy
CCEI	Crown–Crisp Experiential Index
CI	Confidence Interval
IOP	Intraocular pressure
IQR	Interquartile range
OAG	Open-Angle Glaucoma
OCT	Optical coherence tomography
OR	Odds ratio
POAG	Primary Open-Angle Glaucoma
VF	Visual field
